# Human Immunodeficiency Virus (HIV) Risk Behaviors by African American and Puerto Rican Women in the 4^th^ Decade of Life: Substance Use and Personal Attributes

**DOI:** 10.21767/2572-5483.100043

**Published:** 2018-11-29

**Authors:** Jung Yeon Lee, Judith S. Brook, Kerstin Pahl

**Affiliations:** 1 Department of Psychiatry, New York University School of Medicine, USA; 2 Division of Biostatistics, Department of Population Health, New York University School of Medicine, USA; 3 Social Solutions & Services Research, the Nathan S. Kline Institute for Psychiatric Research, USA

**Keywords:** Harlem longitudinal development study, Cannabis use, Self-control, Sexual risk behaviors, Logistic regression analysis

## Abstract

African Americans have the most severe burden of human immunodeficiency virus (HIV) of all racial/ethnic groups in the United States. Also, HIV continues to be a serious threat to the health of the Hispanic/Latino community. For prevention purposes, the present study examined the relationship of both cannabis use and self-control with HIV risk behaviors in a sample of African American and Puerto Rican female adolescents, young adults, and adults. Among the total of 343 female participants, half were African American and the other half were Puerto Rican. Logistic regression analyses were used to examine earlier cannabis use as well as self-control and later HIV risk behaviors. High frequency of cannabis use and high self-control measured at ages 19 to 29 were positively and negatively related to having sexual intercourse with someone they just met at ages 32 to 39. Prevention programs should incorporate the role of cannabis use and low self-control as related to HIV risk behaviors. Our results may have particular utility for designing interventions focused on not only cannabis use (a risk factor) but also self-control (a protective factor) as related to HIV sexual risk behaviors.

## Introduction

According to a report from the Centers for Disease Control and Prevention in 2014 [1], African Americans have the most severe burden of human immunodeficiency virus (HIV) of all racial/ethnic groups in the United States (US). Also, HIV continues to be a serious threat to the health of the Hispanic/Latino community. In 2014, new HIV diagnoses were disproportionately found among African Americans (44%) and Hispanics (24%) in the US [1]. Prior research has focused on HIV risk behaviors in gay and/or bisexual men [2,3]. However, little is known about HIV-relevant behaviors, especially, among minority women in their thirties, who may be at risk for HIV because of their involvement in HIV sexual risk behaviors. Consequently, a greater understanding of the longitudinal predictors of HIV risk behaviors is an important step toward the development of effective prevention programs for women.

Based on the empirical literature [4–6] and Family Interactional Theory (FIT) [7], the present research examined cannabis use as a risk factor and self-control as a protective factor, as well as each of their longitudinal effects on HIV sexual risk behaviors in African American and Puerto Rican women. The HIV sexual risk behavior examined in this study is engaging in sexual intercourse with a man they had just met. The theoretical framework for this study, FIT, is a multidimensional conceptual model of behavior that attempts to explain in part the correlation of substance use (i.e., cannabis use) and personality attributes (i.e., self-control) with HIV sexual risk behavior.

Several investigators suggest that behavioral problems such as substance use correlate with sexual risk behaviors, as shown in data from the Colorado Youth Risk Behavioral Survey and the Monitoring the Future Study [8,9]. More specifically, Nkansah-Amankra et al. [9] found that women who use more illegal substances such as cannabis increased the odds of early sexual initiation. Building on these studies, we examine the linkage between cannabis use and HIV sexual risk behaviors in African American and Puerto Rican women in the fourth decade of life.

Personal attributes such as low self-control are also found to be related to HIV sexual risk behaviors among adolescents and adults [4]. According to this review paper [4], an individual with high self-control is able to tolerate frustration and stress, postpone gratification, modify selfish desires when necessary, and resolve internal conflicts and emotional problems. In contrast, low self-control refers to the inability of the ego to control impulses or tolerate frustration, disappointment, or stress. Here, we highlight the relation of self-control to HIV risk behaviors in women in the fourth decade of life.

The findings regarding a relationship between race/ethnicity and HIV sexual risk behaviors are mixed. African American women at risk have a greater number of HIV-positive or HIV-unknown sex partners as well as concurrent sex partners, and are at greater risk for HIV, compared to Hispanic women [10]. Another study found that Hispanics was associated with higher risk sexual behaviors (e.g., early sexual initiation) and an increased number of sexual partners than African Americans [10].

Based on the theoretical formulation of FIT, we hypothesize the following longitudinal relationships between cannabis use (a risk factor) as well as self-control (a protective factor) and women’s HIV sexual risk behaviors (i.e., sexual intercourse with individuals they just met): a) cannabis use at ages 19, 24, and 29 will be positively associated with HIV sexual risk behaviors at ages 32 to 39; and b) self-control at ages 19, 24, and 29 will be inversely associated with HIV sexual risk behaviors at ages 32 to 39.

## Method

### Participants

This study included 343 female participants (50% African Americans, 50% Puerto Ricans) who completed a seven wave survey administered in the Harlem Longitudinal Development Study. Data on the participants were first collected in 1990 (time 1; T1, N=712) when they were students attending schools in the East Harlem area of New York City. At T1, the questionnaires were administered by study research staff in classrooms without teachers present. At this wave, participants were given an audio version of the questionnaire with which to follow along with the printed version. The mean age of the participants at T1 was 14 years (standard deviation; SD=1.3 years). At time 2 (T2; 1994–1996; N=649), the data were collected by the National Opinion Research Center. The mean age of the participants at this wave was 19 years (SD=1.5 years). At time 3 (T3; 2000 – 2001; N=335-due to budgetary limitations, we took a subsample of T2 participants), the Survey Research Center of the University of Michigan collected the data. The mean age of the participants at T3 was 24 years (SD=1.3 years). Our research group collected the data at time 4 (T4), time 5 (T5), time 6 (T6), and time 7 (T7). At T4 (2004 – 2006; N=498), the mean age of participants was 29 years (SD=1.4 years). At T5 (2007 – 2010; N=492), the mean age of participants was 32 years (SD=1.4 years). At T6 (2011 – 2013; N=405), the average age of the participants was 36 years (SD= 1.4 years). At T7 (2014 – 2016; N=343), the mean age of the participants was 39 years (SD= 1.4 years). The current study included data from the T2, T3, T4, T5, and T7 waves, since data regarding the dependent variable was not available at T6.

The Institutional Review Board (IRB) of the New York University School of Medicine approved the study for T4, T5 and T6, and the IRBs of the Mount Sinai School of Medicine and New York Medical College (our former affiliations) approved the study for the earlier waves. A Certificate of Confidentiality was obtained from the National Institute on Drug Abuse for T1-T7. At each time wave, we obtained informed assent or consent from all of the participants.

### Measures

For a control variable, the participants were asked about their *Race/Ethnicity* at T2 (mean age 19). For the independent variables, the participants were asked about *Cannabis use* (single item) and *Self-control* (a 3 item scale) at T2, T3, and T4 (mean ages 19, 24, and 29) (Cronbach’s alphas for the self-control variables ranged 0.55–0.73). For the dependent variable, the participants responded yes if they have had sexual intercourse with someone they just met at T5 (mean age 32) to T7 (mean age 39).

### Analytic plan

SAS software (9.4) was used to perform simple and multiple logistic regression analyses. In each of the multiple logistic analyses, there were two independent variables including a control variable (i.e., race/ethnicity).

## Results

Among the 343 participants, 24% had sexual intercourse with someone they just met. Table 1 presents the findings from both the simple and multiple logistic regression analyses. The results indicated that individuals who used cannabis at ages 19, 24 and 29 were more likely to have sexual intercourse with someone they just met at age 32 to 39 (Odds Ratio: OR=1.23, p<.05 & Adjusted Odds Ratio: AOR=1.25, p<0.05 at age 19; OR=1.67, p<0.001 & AOR=1.68, p<0.001 at age 24; OR=1.35, p<0.01 & AOR=1.35, p<0.01 at age 29). Higher self-control at ages 19, 24 and 29 (OR=0.88, p<0.05 & AOR=0.88, p<0.05 at age 19; OR=0.82, p<0.01 & AOR=0.82, p<0.05 at age 24; OR=0.83, p<0.01 & AOR=0.83, p<0.01 at age 29) was associated with a decreased likelihood of having sexual intercourse with someone they just met at age 32 to 39.

## Discussion

Overall, our hypotheses indicating the positive association of cannabis use and low self-control with HIV sexual risk behavior were supported. The findings of the present study are in accord with the research reporting the association between cannabis use and behaviors conferring risks for HIV infection [8]. Also, it is consistent with the literature showing that more frequent cannabis use was associated with sexual risk behaviors [9]. In addition, the results showing the association of low self-control with HIV sexual risk behavior was also supported by the recent study using the same sample but using the different dependent variables (e.g., the number of sex partners, alcohol use prior to sexual intercourse, etc.) [11].

The findings of this research emphasize the need for prevention programs focused on cannabis use as well as on self-control in sexually active African American and Puerto Rican women. Based on our findings, intervention programs should focus on decreasing cannabis use in the second and third decades of life. That is, the results of the current study suggest that preventive interventions targeting cannabis use at an earlier stage of development may reduce the risk for HIV related sexual behaviors at later in life. Moreover, interventions might consider attempts at addressing women’s personal attributes such as self-control. For example, nurturing self-control in women beginning in adolescence and young adulthood may have significant effects on their engaging in HIV sexual risk behaviors in adulthood. This may ultimately have implications for later in life.

This research has limitations. First, while our sample included socioeconomic diversity, the participants were all African American and Puerto Rican females. Consequently, we must use caution in considering the generalizability of our findings. Second, there are a number of other factors such as alcohol use and abuse that may influence HIV sexual risk behaviors that were not included in this research. In sum, this research provides an initial picture of how cannabis use and low self-control are related to HIV sexual risk behaviors.

Despite of the limitations, this study has a number of strengths including using prospectively collected data from a longitudinal research project examining the antecedents of HIV sexual risk behaviors. From a developmental perspective, the present study examines African American and Puerto Rican females in late adolescence/young adulthood/adulthood and follows them into the fourth decade of life. Our results may have particular utility for designing interventions focused on not only cannabis use (a risk factor) but also self-control (a protective factor) as related to HIV sexual risk behaviors.

Future studies would benefit from examining how longitudinal trajectories of cannabis use and self-control relate to HIV risk-related behaviors. Research should include additional factors that might mediate the direct effects shown in our model.

## Figures and Tables

**Table 1:** Odds ratios (OR) and adjusted odds ratios (AOR) with 95% confidence interval (CI) of having sexual intercourse with someone they just met. Notes: *p<0.05, **p<0.01, ***p<0.001; NA=Not applicable; for each of the multiple logistic regression analyses, there are two independent variables including race/ethnicity. Data used in this study were collected in the area of New York City during 1995–2016.

Control variables	Having sexual intercourse with someone they just met at age 32 to 39 Or (95% cl)	AOR (95% CI)
**Race/Ethnicity**	0.70 (0.43, 1.13)	NA
**Risk factor-Substance use**		
**Cannabis use at age 19**	1.23 (1.03, 1.48)*	1.25 (1.04, 1.51)*
**Cannabis use at age 24**	1.67 (1.34, 2.07)***	1.68 (1.35, 2.10)***
**Cannabis use at age 29**	1.35 (1.12, 1.62)**	1.35 (1.12, 1.63)**
**Protective factor-Personality attribute**		
**Self-control at age 19**	0.88 (0.77, 0.99)*	0.88 (0.76, 0.99)*
**Self-control at age 24**	0.82 (0.71, 0.95)**	0.82 (0.71, 0.96)*
**Self-control at age 29**	0.83 (0.73, 0.94)**	0.83 (0.73, 0.95)**

## References

[1] https://www.cdc.gov/hiv/group/racialethnic/

[2] Diaz RM (2013) Latino gay men and HIV: Culture, sexuality and risk behaviour. Cul Diver Ethnic Minor Psychol 5: 292–293.

[3] Newcomb ME, Ryan DT, Garofalo R, Mustanski B (2014) The effects of sexual partnership and relationship characteristics on three sexual risk variables in young men who have sex with men. Arch Sex Behav 43: 61–72. 24217953 10.1007/s10508-013-0207-9 PMC3891854

[4] Buhi ER, Goodson P (2007) Predictors of adolescent sexual behavior and intention: A theory-guided systematic review. J Adolesc Health 40: 4–21. 17185201 10.1016/j.jadohealth.2006.09.027

[5] Thamotharan S, Lange K, Herrick A, Sferra M, Fields S, (2014) Adolescent and emerging adult marijuana triers and non-users: The difference in potential HIV risk. Drug Alcohol Depend 140: e223–e224.

[6] Volkow ND, Baler RD, Compton WM, Weiss SR (2014) Adverse health effects of marijuana use. N Engl J Med 370:2219–2227. 24897085 10.1056/NEJMra1402309 PMC4827335

[7] Brook JS, Brook DW, Gordon AS, Whiteman M, Cohen P, (1990) The psychosocial etiology of adolescent drug use: A family interactional approach. Genet Soc Gen Psychol Monogr 116: 111–267. 2376323

[8] http://monitoringthefuture.org/pubs/monographs/vol2_2009.pdf

[9] Nkansah AS, Diedhiou A, Agbanu HL, Harrod C, Dhawan A, (2011) Correlates of sexual risk behaviors among high school students in Colorado: Analysis and implications for school-based HIV/AIDS programs. Matern Child Health J 15: 730–741. 20635195 10.1007/s10995-010-0634-3

[10] McLellan LE, O’Daniels CM, Marks G, Villar LO, Doherty IA, (2012) Sexual risk behaviors among African-American and hispanic women in five counties in the southeastern United States: 2008–2009. Womens Health Issues 22: e9–e18. 21784659 10.1016/j.whi.2011.06.002 PMC4584390

[11] Lee JY, Brook JS, Pahl K, Brook DW (2018) Sexual risk behaviors in African American and Puerto Rican women: Impulsivity and self-control. Prev Med reports 10: 218–220. 10.1016/j.pmedr.2017.09.005 PMC5984213 29868372

